# Enzyme estimates of infarct size correlate with functional and clinical outcomes in the setting of ST-segment elevation myocardial infarction

**DOI:** 10.1186/1468-6708-6-12

**Published:** 2005-08-23

**Authors:** Aslan T Turer, Kenneth W Mahaffey, Dianne Gallup, W Douglas Weaver, Robert H Christenson, Nathan R Every, E Magnus Ohman

**Affiliations:** 1 Department of Internal Medicine, Duke University Medical Center and Duke Clinical Research Institute, Durham, North Carolina, USA; 2 Henry Ford Hospital, Detroit, Michigan, USA; 3 University of Maryland, Baltimore, Maryland, USA; 4 University of Washington, Seattle, Washington, USA; 5 University of North Carolina, Chapel Hill, North Carolina, USA

## Abstract

**Background:**

Cardiac biomarkers are routinely obtained in the setting of suspected myocardial ischemia and infarction. Evidence suggests these markers may correlate with functional and clinical outcomes, but the strength of this correlation is unclear. The relationship between enzyme measures of myocardial necrosis and left ventricular performance and adverse clinical outcomes were explored.

**Methods:**

Creatine kinase (CK) and CK-MB data were analyzed, as were left ventricular ejection fraction (LVEF) by angiogram, and infarct size by single-photon emission computed tomography (SPECT) imaging in patients in 2 trials: Prompt Reperfusion In Myocardial-infarction Evolution (PRIME), and Efegatran and Streptokinase to Canalize Arteries Like Accelerated Tissue plasminogen activator (ESCALAT). Both trials evaluated efegatran combined with thrombolysis for treating acute ST-segment elevation myocardial infarction (STEMI).

**Results:**

Peak CK and CK area-under-the-curve (AUC) correlated significantly with SPECT-determined infarct size 5 to 10 days after enrollment. Peak CK had a statistically significant correlation with LVEF, but CK-AUC and LVEF correlation were less robust. Statistically significant correlations exist between SPECT-determined infarct size and peak CK-MB and CK-MB AUC. However, there was no correlation with LVEF for peak CK-MB and CK-MB AUC. The combined outcome of congestive heart failure and death were significantly associated with CK AUC, CK-MB AUC, peak CK, and peak CK-MB measurements.

**Conclusion:**

Peak CK and CK-MB values and AUC calculations have significant correlation with functional outcomes (LVEF- and SPECT-determined infarct size) and death or CHF outcomes in the setting of STEMI. Cardiac biomarkers provide prognostic information and may serve as valid endpoint measurements for phase II clinical trials.

## Background

There has been considerable interest in validating established cardiac biomarkers as a mechanism for assessing infarct size in clinical practice and as an endpoint in clinical trials. Since assays for creatine kinase (CK) and CK-MB became widely available in the 1970s, attempts have been made to correlate the levels of these biomarkers with outcomes and infarct size using kinetic models and curve-fitting techniques [1-3]. Despite initial enthusiasm, these models were later criticized because of their inability to accurately predict the extent of myocardial necrosis [4-8].

Studies in animals [4,6,9,10] and humans [5,11,12] have shown a significant correlation between CK and CK-MB-derived estimations of myocardial damage after acute myocardial infarction (MI) and the extent of damage at necropsy, albeit in the era prior to reperfusion therapy. Research has suggested that the quantity of cardiac markers released correlates with infarct size and with clinical outcomes such as arrhythmias [13,14], heart failure [13,15-17], and mortality [13,16,18,19] in the setting of both ST-segment elevation myocardial infarction (STEMI) and non-STEMI (NSTEMI). Since cardiac biomarkers are relatively inexpensive to measure and are routinely used in clinical practice, they are an attractive tool for easily determining infarct size and gathering prognostic information.

Little has been done to determine how the different methods used to assess infarct size in acute MI patients treated with reperfusion therapy correlate. Improving our understanding of the methods used to measure infarct size and clarifying the association between infarct size and outcomes can be of tremendous value to clinical trials, particularly in the early evaluation of new therapies or interventions.

The aims of the current analyses were to 1) determine whether enzyme-determined infarct size would correlate to the degree of infarction measured by single-photon emission computed tomography (SPECT) imaging and to left ventricular ejection fraction (LVEF) measured by LV angiography, and 2) assess whether the amount of enzyme released could be used to predict clinical outcomes in patients treated with thrombolysis. For this analysis, data from the Efegatran and Streptokinase to Canalize Arteries Like Accelerated Tissue plasminogen activator (ESCALAT) [20] and Prompt Reperfusion In Myocardial-infarction Evolution (PRIME) [21] trials were used.

## Methods

### The trials

The ESCALAT trial has been published [20]. In brief, ESCALAT was a randomized, dose-finding study in which intravenous (IV) efegatran sulfate (in 1 of 4 doses) plus streptokinase, or IV heparin plus accelerated tissue plasminogen activator (t-PA) was given to patients (N = 245) presenting with acute STEMI. Trial participants were between 21 and 75 years old, had ischemic chest pain for ≥ 30 minutes associated with ST-segment elevation of ≥ 0.1 mV in 2 or more contiguous electrocardiographic leads, and onset of symptoms within 12 hours of planned treatment. The primary endpoint of infarct-related artery patency was assessed by angiography at 90 minutes.

In the PRIME trial [21], patients (N = 336) with acute MI were randomly assigned to accelerated t-PA and either IV efegatran sulfate (in 1 of 5 doses) or IV heparin. In PRIME, inclusion and exclusion criteria were similar to those for ESCALAT [20,21]. The study protocol required coronary angiography 90 minutes after start of t-PA therapy and repeat angiography 5 to 7 days later.

### SPECT and left ventricular (LV) functional assessments

SPECT quantification of infarct size was performed 5 to 10 days after randomization per the ESCALAT protocol. SPECT images were evaluated in blinded fashion by a central core laboratory (Georgetown University, Washington, DC) using previously described methodologies [22].

In the PRIME trial, patients who did not have percutaneous coronary intervention or bypass surgery were to have repeat coronary angiography 5 to 7 days after enrollment or prior to hospital discharge, whichever occurred first. All angiograms were reviewed by an independent evaluator, blinded to treatment assignment, at a central core laboratory (Cleveland Clinic, Cleveland, Ohio). Angiograms were assessed for vessel patency, Thrombolysis In Myocardial Infarction (TIMI) flow grades, and LV function.

### Biomarker determination of infarct size

**Figure 1:**
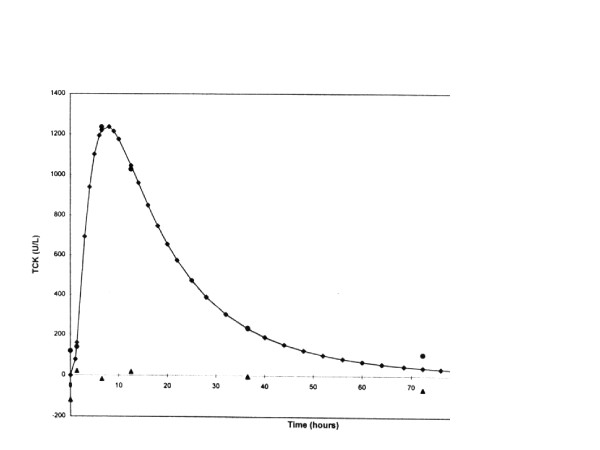
**Log normal plot of total CK data for a typical patient**. The connected points are from curve fitting, the solid circles are the actual measured points, and the solid triangles (along x-axis) are the residuals. In this case, the measured 8-hour point correlates well with the calculated peak for the curve. **CK**: Creatine kinase, **TCK**: Total CK.

In both the ESCALAT and PRIME trials, CK and CK-MB levels were to be drawn at baseline (time of enrollment) and at 6, 12, 24, 36, and 72 hours after enrollment. All samples were analyzed at a central core laboratory (University of Maryland, College Park, MD), and the evaluator was blinded to patient information. Cardiac biomarker curves were then plot-fitted by the method proposed by Vollmer and colleagues [23]. Area-under-the-curve (AUC) and peak biomarker levels were derived for both CK and CK-MB from these curve-fit data (Figure 1).

### Clinical outcomes

Clinical outcomes of death or congestive heart failure (CHF) that occurred during the index hospitalization were reported by the investigator with standard data collection tools without adjudication using standard definitions.

### Statistical analysis

Statistical analyses were performed with SAS software (version 6, SAS, Inc., Cary, North Carolina) on the UNIX system. Continuous variables were summarized using medians and interquartile ranges whereas categorical variables were summarized as frequencies and percentages.

**Figure 2:**
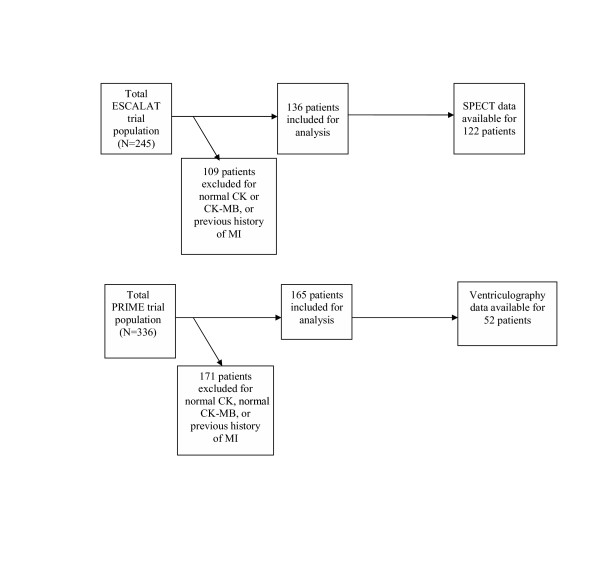
**PRIME and ESCALAT study populations**. **CK**: Creatine kinase, **MI**: Myocardial infarction. **SPECT**, Single-photon emission computed tomography.

Patients from both trials were excluded from analyses if they had a history of MI or no elevation of CK or CK-MB due to confounding. Within the ESCALAT trial, after these exclusions, the study size was 136 patients, representing 56% of the original study population. From the PRIME trial, 165 patients were included in the analysis after exclusions, accounting for 49% of the original trial population (Figure 2).

Spearman rank correlations were used to determine associations between the cardiac biomarker data and size of infarct as measured by SPECT for the ESCALAT trial and LVEF for the PRIME trial. Wilcoxon rank sum tests were used to determine differences in continuous variables between PRIME and ESCALAT.

## Results

### Baseline demographics

The baseline demographics were similar for the ESCALAT and PRIME trial populations with respect to age, sex, race, and history of smoking. None of the patients had a history of MI as part of the inclusion criteria for this analysis.

### Clinical outcomes

There were no significant differences in death or CHF during the index hospitalization between the treatment groups in either trial. There were also no statistical differences between the trials with respect to CK and CK-MB data (Table 1); therefore, the trials were combined for further analysis of enzyme release. Table 1 shows the infarct size assessments by peak CK, peak CK-MB, CK-AUC, CK-MB AUC, LVEF, and SPECT data.

**Table 1:** ESCALAT and PRIME trials: assessments of myocardial damage

	**ESCALAT n = 136**	**PRIME n = 165**	***P *value**
**Peak CK**			
n	127	158	
Median (25th, 75th)	1790.0 (682, 2580)	1730.0 (931, 2850)	0.6718
**Peak CK-MB**			
n	129	161	
Median (25th, 75th)	209 (82, 340)	207 (101, 362)	0.6239
**CK-MB AUC**			
n	129	161	
Median (25th, 75th)	3887.0 (1617, 6099)	3891.0 (2035, 6746)	0.1588
**CK AUC**			
n	127	158	
Median (25th, 75th)	50960 (22950, 79620)	51295 (29930, 87390)	0.1801
**LVEF (%)**			
n	n/a	52	
Median (25th, 75th)	n/a	58.0 (50, 66)	n/a
**SPECT (% infarcted**)			
n	122	n/a	
Median (25th, 75th)	13.0 (4, 24)	n/a	n/a

*P *values obtained using Wilcoxon rank sum test.

**AUC: **Area-under-the-curve, **CK: **creatine kinase, **ESCALAT: **Efegatran and Streptokinase to Canalize Arteries Like Accelerated Tissue plasminogen activator, **LVEF: **left ventricular ejection fraction, **PRIME: **Prompt Reperfusion In Myocardial infarction Evolution, **SPECT: **single-photon emission computed tomography

### Correlation of enzymes and SPECT

In ESCALAT, no differences in infarct size by SPECT imaging, CK-AUC, or CK-MB AUC were observed between the efegatran-plus-streptokinase- and heparin plus t-PA-treated groups. Therefore, all treatment groups were combined for these analyses. The median (25th, 75th) time to SPECT was 6 days [5,16].

**Figure 3:**
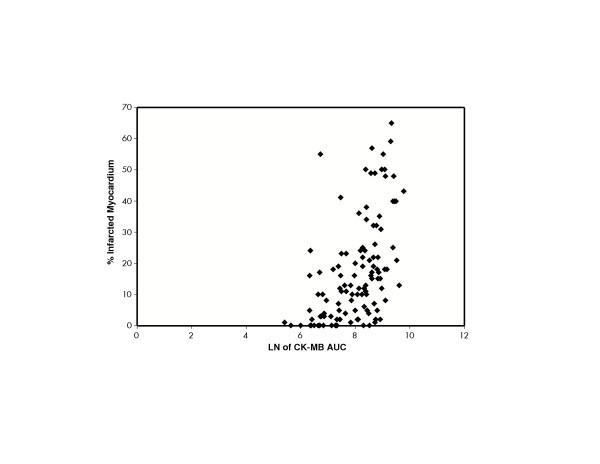
**Natural log of peak CK-MB and percent infarcted myocardium (SPECT)**. **AUC**: Area-under-the-curve, **CK**: Creatine kinase, **LN**: log normal, **SPECT**: Single-photon emission computed tomography.

A scatter-plot of CK-MB AUC and SPECT infarct size is shown in Figure 3. Statistically significant positive correlations between the SPECT-determined infarct size and the peak values for CK (*r *= 0.65, *P *< 0.0001) and CK-MB (*r *= 0.64, *P *< 0.0001) were observed. SPECT infarct size also correlated with the AUC measurements for both CK (*r *= 0.63, *P *< 0.0001) and CK-MB (*r *= 0.58, *P *< 0.0001) (Table 2).

**Table 2:** ESCALAT and PRIME trials: correlations of enzymes with LVEF- and SPECT-determined infarct size

	**Peak CK**	**Peak CK-MB**	**CK AUC**	**CK-MB AUC**
**LVEF**	*r *= -0.30	*r *= -0.26	*r *= -0.28	*r *= -0.22
	*P *= 0.0354	*P *= 0.0599	*P *= 0.0508	*P *= 0.1117
	n = 50	n = 51	n = 50	n = 51
**SPECT**	*r *= 0.65	*r *= 0.64	*r *= 0.63	*r *= 0.58
	*P *< 0.0001	*P *< 0.0001	*P *< 0.0001	*P *< 0.0001
	n = 115	n = 116	n = 115	n = 116

**AUC: **Area-under-the-curve; **CK: **creatine kinase, **ESCALAT: **Efegatran and Streptokinase to Canalize Arteries Like Accelerated Tissue plasminogen activator, **LVEF: **left ventricular ejection fraction, **PRIME: **Prompt Reperfusion In Myocardial infarction Evolution, **SPECT: **single-photon emission computed tomography

### Correlation of enzymes and LVEF

In PRIME, repeat angiography with LVEF assessment was repeated after a median (25th, 75th) of 5 days [4,6] from thrombolysis. A statistically significant negative correlation existed between LVEF measured at repeat angiography and the peak levels of CK (*r *= -0.30, *P *= 0.035). Borderline significant correlations were observed between LVEF and CK-AUC (*r *= -0.28, *P *= 0.051). The negative correlation between LVEF and peak CK-MB (*r *= -0.26, *P *= 0.06) as well as between LVEF and CK-MB AUC (*r *= -0.22, *P *= 0.11) did not achieve statistical significance (Table 2).

### Association between enzymes and outcomes

**Figure 4:**
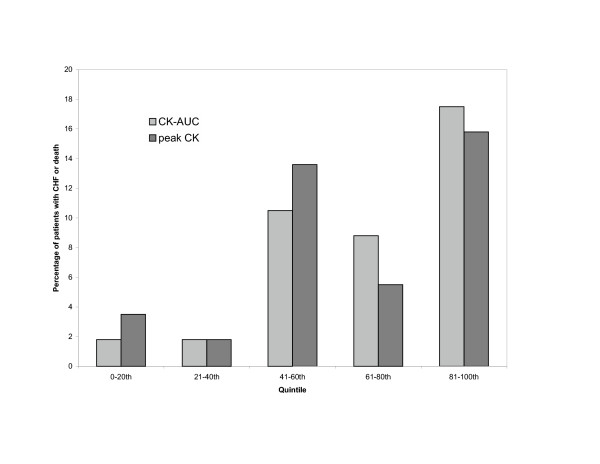
**Incidence of new CHF and death by CK-AUC and peak CK measurements, according to quintiles**. **AUC**: Area-under-the-curve, **CHF**: congestive heart failure, **CK**: Creatine kinase.

**Figure 5:**
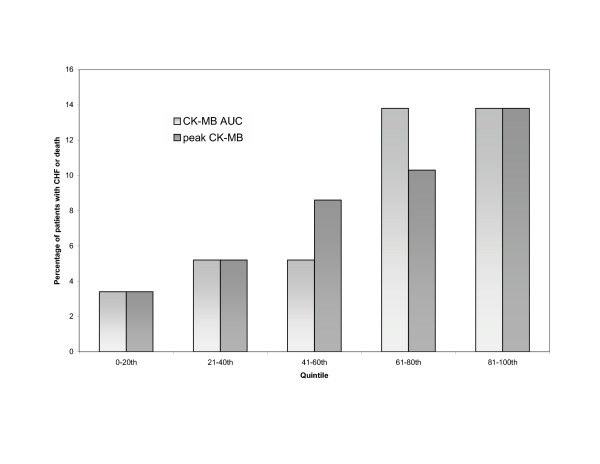
**Incidence of new CHF and death by CK-MB AUC and peak CK-MB measurements, according to quintiles**. AUC: Area-under-the-curve, CHF: congestive heart failure, CK: Creatine kinase.

The total number of events in each trial is shown in Table 3. Figure 4 displays a graph of death/CHF and peak CK/CK-AUC quintiles. Figure 5 is a similar graph of death/CHF and peak CK-MB /CK-MB AUC quintiles. The Wilcoxon rank sum test resulted in a statistically significant association between death/CHF and peak CK (*P *= 0.007) and AUC (*P *= 0.001) measurements; there was also an association between death/CHF and peak CK-MB (*P *= 0.021) and AUC (*P *= 0.005) measurements.

**Table 3:** ESCALAT and PRIME trials: clinical outcomes

	**ESCALAT n = 136**	**PRIME n = 165**
Death	2 (1.5)	5 (3.0)
New CHF	Not collected	21 (12.7)
Death/new CHF	2 (1.5)	22 (13.3)

Values are n (%)

**CHF: **Congestive heart failure, **ESCALAT: **Efegatran and Streptokinase to Canalize Arteries Like Accelerated Tissue plasminogen activator, **PRIME: **Prompt Reperfusion In Myocardial infarction Evolution

## Discussion

This study showed a significant correlation between infarct size as assessed by SPECT and infarct size as determined by CK and CK-MB release using AUC or peak values. Weaker correlations were observed between infarct size measured by cardiac biomarker assessments and LVEF determined by angiography. Patients with larger release of CK or CK-MB had an increased risk of death and/or CHF.

These findings are consistent with earlier studies [12,24-33] that have also examined the correlation between CK and CK-MB and SPECT-determined infarct size. However, the majority of these analyses were done in the era prior to reperfusion therapy with fibrinolytic therapy or percutaneous coronary intervention. Therefore, the current analysis is important for trials being planned in the STE AMI population with contemporary therapies.

Attempts have been made to correlate CK- and CK-MB-based infarct measurements with LV function, with conflicting results. EF has been variably correlated to CK-MB AUC [17,34], peak CK-MB [17,35], CK AUC [15,36], and peak CK [36] in several angiographic studies, as well as in 1 radionucleotide study involving patient numbers similar to those in our study. Although the data confirm a significant association between LVEF determined by angiography and peak CK, the Spearman coefficient revealed only a weak relationship. This may have been in part due to small sample sizes.

The relatively poor correlation noted here between angiographic and enzymatic methods of infarct sizing might be due to post-infarct stunning, leading to an artifactually low LVEF; earlier clinically or electrocardiographically silent MIs contributing to LV dysfunction attributed to the index event; site of infarct (inferior vs. anterior); and the degree of right ventricular involvement. There also appears to be wide variability in the published Spearman coefficients on enzyme/LVEF correlations, with the current data falling at the lower end of the previously published ranges. These discrepant results might be explained by a number of factors, including variations in enzyme curve measurements; inter- and intra-study variability of LVEF measurements; timing of catheterization relative to MI; differences in treatment allotments; the year of study, which has implications for the availability of reperfusion and improvements in coronary care; and publication bias, with nonsignificant or weak association preferentially not being reported in the literature.

The ability of cardiac enzymes to predict clinical outcomes would enhance confidence in the use of this assessment in phase II clinical trials but would not exclude the need to conduct definitive trials designed to assess clinical outcomes directly. An early study of patients presenting with acute MI prior to the era of widespread revascularization showed a correlation between peak CK and CK-AUC and arrhythmias, pulmonary edema, and higher pulmonary artery wedge pressure [37]. Subsequently, in the Global Utilization of Streptokinase and Tissue plasminogen activator for Occluded coronary arteries (GUSTO-I) enzyme substudy [38], a 12% decrease in infarct size (as measured by α-hydroxybutyrate dehydrogenase), with t-PA (compared with streptokinase) corresponded to the 14% relative mortality reduction in the trial overall [39]. More recently, analysis from the Thrombolysis and Angioplasty in Myocardial Infarction (TAMI-7) trial of accelerated alteplase in patients presenting with ST-segment elevations showed a clear correlation between CK-MB peak and AUC values and development of CHF and a composite of CHF and death [17]. Similarly, data from the Platelet glycoprotein IIb/IIIa in Unstable angina: Receptor Suppression Using Integrilin Therapy (PURSUIT) trial showed a graded risk of death at 30 days and 6 months based on peak CK and CK-MB in NSTEMI [19]. In keeping with prior studies, we were able to demonstrate a graded risk of adverse cardiovascular outcomes (defined as new CHF and death) with a statistically significant increase of events in patients in higher quintiles of peak and AUC measurements for both CK and CK-MB.

The strengths of the present study are that the data were collected in the context of 2 randomized, controlled trials with strict adherence to serial biomarker assessments, analyses by a central core laboratory, and sophisticated curve-fitting techniques. Most of the noted previous studies used the modified formula proposed by Roberts et al [2] in 1975, to calculate the cumulative enzyme release. For this study, in contrast, a log normal function was used to correlate AUC values and to curve-fit in order to find peak enzyme levels; more recent data show that the log normal function best approximates the true enzyme release curve and peak values [23]. This method is easy to use and requires less sampling than other methods of curve fitting. The log normal function has obvious advantages over older methods and a clear role in data collection for prospective studies of infarction.

### Limitations

Several weaknesses should be acknowledged. Sample sizes from the 2 trials were relatively small. The ventriculographic and scintigraphic data were incomplete, which may have implications if the missing data were significantly different from that reported here. Also, to more accurately assess the damage done by the index infarction, this analysis included only patients presenting with their first MI. This factor may affect the generalizability of the results. Although plot-fitting appears to be the most accurate method of determining enzyme peaks and integrating areas given current routine practices in measuring cardiac markers, it is possible that the sampling frequency used misrepresented the true nature of the enzyme curves. Finally, cardiac biomarkers, though useful, still represent surrogate measurements of clinical outcomes and need to be used with caution in the evaluation of new therapies, because a new treatment that has had no effect on a surrogate measurement (such as CK or CK-MB) might still have an important treatment effect.

Given the small sample size, we were not able to rigorously assess the potential impact of infarct location on the relationships observed. The contribution of right ventricular infarct to CK-MB release and the correlation of this release to assessment of left ventricular function could confound these findings.

### Implications

Given their affordability and routine availability, cardiac biomarkers remain an attractive tool for measuring myocardial damage and determining prognosis. Cardiac enzyme markers continue to be used as endpoints in clinical trials, such as the Complement And ReDuction of INfarct size after Angioplasty or Lytics (CARDINAL) program [40,41] and the Limitation of Myocardial Infarction Following Thrombolysis in Acute Myocardial Infarction (LIMIT AMI) [42] trials. These results support the use of biomarker-based infarct sizing in the evaluation of therapeutics, treatment effects, and outcomes.

An attractive alternative in early drug development is to continue to combine a variety of measures of efficacy, such as cardiac enzymes, ECG analysis, SPECT, and angiography, to develop a "biomarker array." If multiple validated and significant prespecified outcomes are analyzed in the context of phase II clinical research, fewer patients would potentially need to be enrolled to discern differences in treatment effects between trial arms. This analysis substantiates the concept that routine cardiac markers can predict infarct size and clinical outcomes and may therefore act as a valid endpoint in phase II trials of acute MI therapy.

## Conclusion

Serial cardiac biomarker assessment and curve-fitting techniques can be used to determine infarct size with statistically significant correlation to SPECT imaging. Weaker correlations were observed with LVEF by angiography. Death and CHF outcomes were associated with larger infarct size as determined by biomarkers. These data support the use of biomarker-determined infarct size as a potential endpoint in phase II clinical trials.

## Abbreviations

AUC, area-under-the-curve

CHF, congestive heart failure

CK, creatine kinase

LVEF, left ventricular function

MI, myocardial infarction

NSTEMI non-ST-segment myocardial infarction

SPECT, single-photon emission computed tomography

STEMI, ST-segment myocardial infarction

t-PA, tissue plasminogen activator

## Competing interests

The authors have no competing interests to declare.

The PRIME trial was funded by grants from Centocor, Inc., Malvern, Pennsylvania, and Eli Lilly and Company, Indianapolis, Indiana. The ESCALAT trial was sponsored by Lilly Research Laboratories, Indianapolis, Indiana; Eli Lilly Canada, Inc., Scarborough, Ontario, Canada; and LillyResearch Center Ltd., Surrey, United Kingdom.

## Authors' contributions

ATT served as a primary author. KWM served as a primary author and provided critical review of manuscript. DG provided statistical analyses. WDW provided critical review of manuscript. RHC provided critical review of manuscript. NRE provided critical review of manuscript. EMO provided critical review of manuscript. All authors have given final approval of the version to be published.
